# Encapsulation of a Ru(II) Polypyridyl Complex into Polylactide Nanoparticles for Antimicrobial Photodynamic Therapy

**DOI:** 10.3390/pharmaceutics12100961

**Published:** 2020-10-13

**Authors:** Nancy Soliman, Vincent Sol, Tan-Sothea Ouk, Christophe M. Thomas, Gilles Gasser

**Affiliations:** 1 Institut de Recherche de Chimie Paris, CNRS, Chimie ParisTech, PSL University, 75005 Paris, France; nancy.soliman@chimieparistech.psl.eu; 2 Laboratory for Inorganic Chemical Biology, Institute of Chemistry for Life and Health Sciences, CNRS, Chimie ParisTech, PSL University, 75005 Paris, France; 3 Laboratoire PEIRENE, Limoges University, EA 7500, 123 Avenue Albert Thomas, 87060 Limoges, France; vincent.sol@unilim.fr

**Keywords:** antimicrobial photodynamic therapy, nanoparticles, photodynamic inactivation, polylactide, ruthenium complexes

## Abstract

Antimicrobial photodynamic therapy (aPDT) also known as photodynamic inactivation (PDI) is a promising strategy to eradicate pathogenic microorganisms such as Gram-positive and Gram-negative bacteria. This therapy relies on the use of a molecule called photosensitizer capable of generating, from molecular oxygen, reactive oxygen species including singlet oxygen under light irradiation to induce bacteria inactivation. Ru(II) polypyridyl complexes can be considered as potential photosensitizers for aPDT/PDI. However, to allow efficient treatment, they must be able to penetrate bacteria. This can be promoted by using nanoparticles. In this work, ruthenium-polylactide (**RuPLA**) nanoconjugates with different tacticities and molecular weights were prepared from a Ru(II) polypyridyl complex, **RuOH**. Narrowly-dispersed nanoparticles with high ruthenium loadings (up to 53%) and an intensity-average diameter < 300 nm were obtained by nanoprecipitation, as characterized by dynamic light scattering (DLS). Their phototoxicity effect was evaluated on four bacterial strains (*Staphylococcus aureus, Staphylococcus epidermidis*, *Escherichia coli* and *Pseudomonas aeruginosa*) and compared to the parent compound **RuOH**. **RuOH** and the nanoparticles were found to be non-active towards Gram-negative bacterial strains. However, depending on the tacticity and molecular weight of the **RuPLA** nanoconjugates, differences in photobactericidal activity on Gram-positive bacterial strains have been evidenced whereas **RuOH** remained non active.

## 1. Introduction

With an increasing number of microorganisms showing resistance to antibiotics, we are now stepping into the “post-antibiotic” era—as World Health Organization (WHO) first reported in 2014—in which common and minor infections can (once again) be fatal [1]. This major health concern has led to the development of new antimicrobial treatments, including antimicrobial photodynamic therapy (aPDT) [2,3,4]. aPDT is a two-stage procedure involving the administration of a photosensitizer (PS) followed by local irradiation of light of a specific wavelength. Upon light irradiation, the PS is promoted to an excited state from which it can interact with its biological surroundings to form reactive oxygen species (ROS), including the highly cytotoxic singlet oxygen (^1^O_2_). This technique was first introduced in 1900 by Oscar Raab when he reported the toxicity of acridine upon sunlight exposure towards *Paramecia*, a type of microorganism [5]. Research on aPDT has slowed down since the revolutionary discovery of penicillin in 1928, which marked the beginning of the antibiotic area. However, it is slowly regaining interest among the scientific community to tackle the limitations encountered with currently used antibiotics. One of the limitations is the development of bacterial resistance due to the misuse and overuse of antibiotics. The growing interest in aPDT mainly relies on its multi-target nature involving the rapid and effective action of ROS, which makes it less prone to resistance, unlike conventional antimicrobial treatments [6].

The development of alternative antimicrobial treatment modalities comes with the development of new classes of antimicrobials like metal complexes [7,8,9]. Among them, Ru(II) complexes, including Ru(II) polypyridyl complexes, hold great promise to inactivate and eradicate pathogenic microorganisms including bacteria, fungi, and viruses [10,11,12]. Some mono- and poly-nuclear Ru(II) complexes have already been reported to effectively reduce the viability of different bacterial strains [13,14,15,16,17,18,19,20,21,22,23,24,25]. However, research on the antimicrobial activity of Ru(II) polypyridyl complexes upon light irradiation is still in its infancy. Only a few articles in the field of aPDT reported on the photo-inactivation of microorganisms by these types of complexes [26,27,28,29], taking benefits from the large variety of Ru(II) polypyridyl complexes developed for cancer treatment [30]. These studies highlighted the importance of lipophilicity, charge, and charge separation of molecules on antimicrobial activity.

To allow efficient aPDT, PSs must be able to bind and penetrate microbial cells. Binding and uptake of PS by the microbial cells can be improved by using polymeric nanoparticles (NPs), which not only allow the delivery of photosensitizers to bacteria but also, in some cases, increase the ^1^O_2_ quantum yield production by preventing the quenching effects that some PSs endure in an aqueous medium. So far, this strategy has only been used with organic photosensitizers [31,32,33,34]. There are two ways to prepare NPs for PS delivery: (i) covalent conjugation where a chemical bond is used to attach the PS to a constituent of the nanostructure and (ii) physical encapsulation. Although the physical encapsulation is widely used, it suffers from strong limitations including burst release and poor drug loading which can be overcome using the covalent encapsulation.

**Scheme 1:**
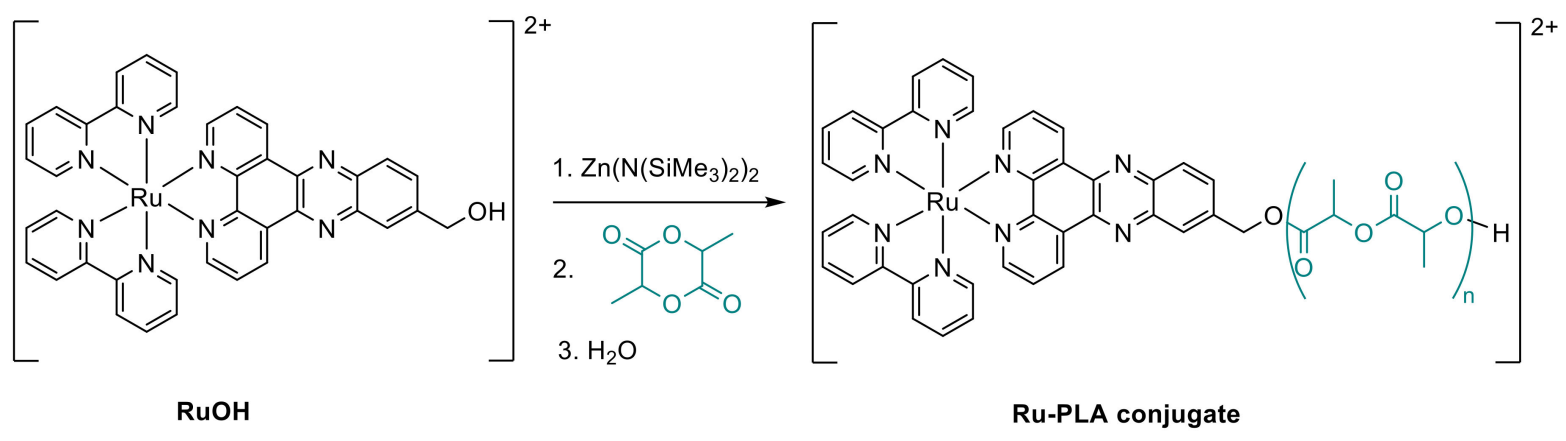
Synthesis of **RuPLA** from **RuOH**.

In this regard, we hypothesized that [Ru(bipy)_2_-dppz-7-hydroxymethyl][PF_6_]_2_ (bipy = 2,2′-bipyridine, dppz = dipyrido[3,2-*a*:2;2′,3′-*c*]phenazine) (**RuOH**) [35], a previously described Ru(II) polypyridyl complex, could be a potential antimicrobial agent when associated with an appropriate drug carrier. As already demonstrated by our groups [36], the lipophilicity of this complex can be increased by its conjugation to the end-chain of a hydrophobic polylactide (PLA) via the drug-initiated ring-opening polymerization (ROP) of lactide (LA), resulting in ruthenium-polylactide (**Ru-PLA**) conjugates that can be formulated into sub-300 nm NPs by nanoprecipitation (Scheme 1). These **Ru-PLA** nanoconjugates were characterized by superior photophysical properties, including luminescence and enhanced ^1^O_2_ generation due to a lower amount of quenching effects in aqueous media compared to **RuOH** alone, and a moderate phototoxicity against cancerous human cervical carcinoma (HeLa) cells. An IC_50_ as low as 4.4 μM and a phototoxicity index (PI) up to 11 were unveiled. These results prompted us to investigate their antimicrobial activity against four bacterial strains, two of which are Gram-positive (*Staphylococcus aureus* and *Staphylococcus epidermidis*) and the other two are Gram-negative (*Escherichia coli* and *Pseudomonas aeruginosa*) bacteria. To the best of our knowledge, this is the first time that Ru(II) polypyridyl complexes encapsulated in polymer NPs have been used in aPDT.

## 2. Materials and Methods

### 2.1. Materials

The preparation of **RuPLA** conjugates were carried out under a purified argon atmosphere using Schlenk techniques or a glovebox (Jacomex, Lyon, France) (<1 ppm O_2_, <2 ppm H_2_O) and then formulated into NPs by nanoprecipitation at 20 °C as previously reported by our groups [36]. 

### 2.2. Instrumentation and Methods

The nanoparticle intensity-average diameters *D*_z_ and the polydispersity index (PdI) were determined by dynamic light scattering (DLS) using a ZetaSizer (Nano ZS, Malvern Instruments, Worcestershire, UK) with a scattering angle of 173° at a temperature of 25 °C with an equilibrium time of 120 s. The zeta-potential (mV) was measured at 25 °C after dilution with 1 mM NaCl using the Smoluchowski equation.

### 2.3. Bacterial Strains and Conditions of Culture

Gram-positive (*Staphylococcus aureus* CIP76.25 and *Staphylococcus epidermidis* CIP109.562) and Gram-negative (*P. aeruginosa* CIP76110 and *E. coli* CIP54.8T) bacterial strains were obtained from Collection Institut Pasteur (CIP, Institut Pasteur Paris, France). These strains were cultured in liquid tryptic soy broth (pancreatic casein extract 17 g/L, soy flour papaic digest 3 g/L, dextrose 2.5 g/L, NaCl 5 g/L, and K_2_HPO_4_ 2.5 g/L) and incubated overnight at 37 °C under aerobic conditions.

### 2.4. Bacteria Photoinactivation

Fresh solutions of **RuOH** and NPs were directly dissolved into Phosphate-Buffered Saline 1X (PBS1X, without Ca^2+^ and Mg^2+^) at a concentration of 100 µM. From these mixtures, 50 µL of serial dilutions (100 μM down to 78 nM) were transferred into two 96-well plates (BD Falcon, Le Pont de Claix, France). An amount of 50 µL of a bacterial culture at a concentration of 4 × 106 UFC/mL was deposited in each well. Bacteria were put in contact with each compound for 4 h at 37 °C in the dark. Subsequently, the 96-well plates (Thermo Scientific, Illkirch, France) were irradiated with LED visible light (device built in the lab) (4.83 mW/cm^2^) for a total fluence of 25 J/cm^2^. Controls consisting of 96-well plates were prepared in the same conditions but kept in the dark. After irradiation, 100 µL of two-fold-concentrated culture media were added in each well and the 96-well plates were incubated overnight 37 °C under aerobic conditions. The lowest concentration of each nanoparticle that prevented bacterial growth was considered as the minimum inhibitory concentration (MIC) of that nanoparticle. Bacterial count was performed after a 10-fold serial dilution of each well where an absence of bacterial growth was observed. Each dilution was spread on tryptic soy agar plates using an automatic plater (easySPIRAL, Interscience, Saint Nom La Bretêche, France). After incubation at 37 °C for 24 h, colonies were counted to determine total colony-forming units (CFU) per milliliter (CFU/mL). The minimum bactericidal concentration (MBC) corresponds to the concentration of the active compound at which 99.99% of the bacteria have been killed (i.e., 4 log reduction compared to the untreated control). A total of three independent experiments were performed with each strain.

### 2.5. Effects of Light Fluence

To observe the effects of light fluence (J/cm^2^) on the survival rate of each strain, bacteria (2 × 106 UFC/mL) were incubated 4 h at 37 °C in the dark with 100 µL of the different solutions of NPs at a concentration of 50 µM. Then, bacteria were collected by centrifugation (10.000 rpm, 5 min) and washed twice with PBS (1× without Ca^2+^ and Mg^2+^). Washed bacteria were resuspended in PBS, transferred into a 96-well plate and irradiated by white LED (4.83 mW/cm^2^). Survival rates, calculated in relation to the initial count of each bacterial culture, were plotted by the function of cumulative fluence (J/cm^2^). Three independent experiments were performed with each strain.

### 2.6. Flow Cytometry

For each bacterial strain, 108 CFU were put in contact with 500 µL of each nanoparticle (10 μM in PBS) for 4 h at 37 °C in the dark. Then, bacteria were retrieved by centrifugation and washed with sterile PBS. Bacteria were resuspended in 500 μL of 1× PBS after washing step. Before analysis by flow cytometry, 10 μL of propidium iodide (PI, 0.5 mg/mL) was added to the bacterial suspension. Fluorescence emissions were analyzed with a BD FACSAria III cell sorter (BD Biosciences, Le Pont de Claix, France). Bacterial cells were excited by two lasers. NPss were excited with a 405-nm violet laser and the fluorescence emitted was detected with a BV650 (670/30 nm) filter. PI was excited with a 561-nm yellow-green laser and the fluorescence emitted was detected with a BV605 (610/20 nm) filter. Overall, 10,000 events were counted.

## 3. Results and Discussion

### 3.1. Preparation of **RuPLA** Nanoconjugates

**RuOH** was conjugated to FDA-approved polylactide via the drug-initiated ROP of LA to give **RuPLA** conjugates with different microstructures and chain lengths (i.e., 2000, 4000 and 7000 g/mol), as previously reported (Table 1) [36]. These conjugates were prepared either from a racemic mixture of d,l-lactide or from the enantiopure d-lactide or l-lactide. Polymers derived from d,l-lactide yielded an amorphous polymer as a result of the random sequence of d and l-units along the polymer backbone, whereas polymers derived from the enantiopure monomers yielded semi-crystalline isotactic polymers PLLA and PDLA with a melting temperature *T*_m_ around 140 °C. Mixing two isotactic polylactides of opposite configurations (PLLA and PDLA) at an equimolar ratio allowed the formation of a stereocomplex characterized by superior physical properties, in particular thermal properties, with a *T*_m_ 60 °C higher than that of the respective homochiral polymers, in accordance with what has already been reported in the literature [37,38,39]. These conjugates were then formulated into reproducible and narrowly dispersed NPs with an intensity-average diameter Dz lower than 300 nm and a polydispersity index (PdI) around 0.2. The reproducibility of the preparation with respect to particle size was investigated by analyzing three independent batches (Figures S1–S4). The surface charge of NPs was also investigated by zeta-potential (ζ, mV) measurements. Depending on the microstructure of the **RuPLA** nanoconjugates, the zeta potential was different. While stereocomplex NPs (namely, **NPs3** and **NPs5**) possess a zeta potential below 10 mV, atactic NPs (namely, **NPs1**, **2** and **4**) have a positive zeta potential ca. 30 mV, suggesting that **RuOH** might be present on the surface of the atactic NPs.

The ruthenium loading % **RuOH** was determined by ^1^H NMR spectroscopy and confirmed after formulation by UV-vis spectrophotometry using the absorption peak of **RuOH** at 450 nm.

### 3.2. Biological Evaluation of **RuPLA** Nanoconjugates

The photobactericidal activity of **RuOH** and NPs was established through biological assays against four bacterial strains, two Gram-positive strains (*S. aureus* and *S. epidermidis*) and two Gram-negative strains (*E. coli*, and *P. aeruginosa*), by determining their minimum inhibitory concentrations (MIC) and minimum bactericidal concentrations (MBC). While MIC assays determine the lowest concentration of an antimicrobial agent that prevents visible growth of a microorganism, MBC assays determine the lowest concentration that reduces the viability of the initial bacterial inoculum by ≥99.9% which represents a three-logarithmic decrease. Each experiment was realized in triplicate and repeated three times. 

**RuOH** and all NPs were ineffective against both Gram-negative strains at 50 μM with or without light irradiation, indicating that their MIC and MBC are higher than 50 μM. 

On Gram-positive strains, **RuOH** and all NPs were ineffective under dark conditions at 50 µM. However, after light irradiation with LED visible light (4.83 mW/cm*^2^*) for a total fluence of 25 J/cm*^2^*, **NPs1, 3** and **5** showed a photobactericidal activity against *S. aureus* and *S. epidermidis* (Table 2). The most active NPs are **NPs3** and **NPs5**, the two stereocomplex ones, with a photobactericidal activity at 12.5 µM. We can therefore conclude that NPs increase the photobactericidal capacity of **RuOH**. Indeed, this enhancement is probably related to the increased hydrophobicity rendered possible by the PLA chain and the microstructure of the NPs.

**Figure 1:**
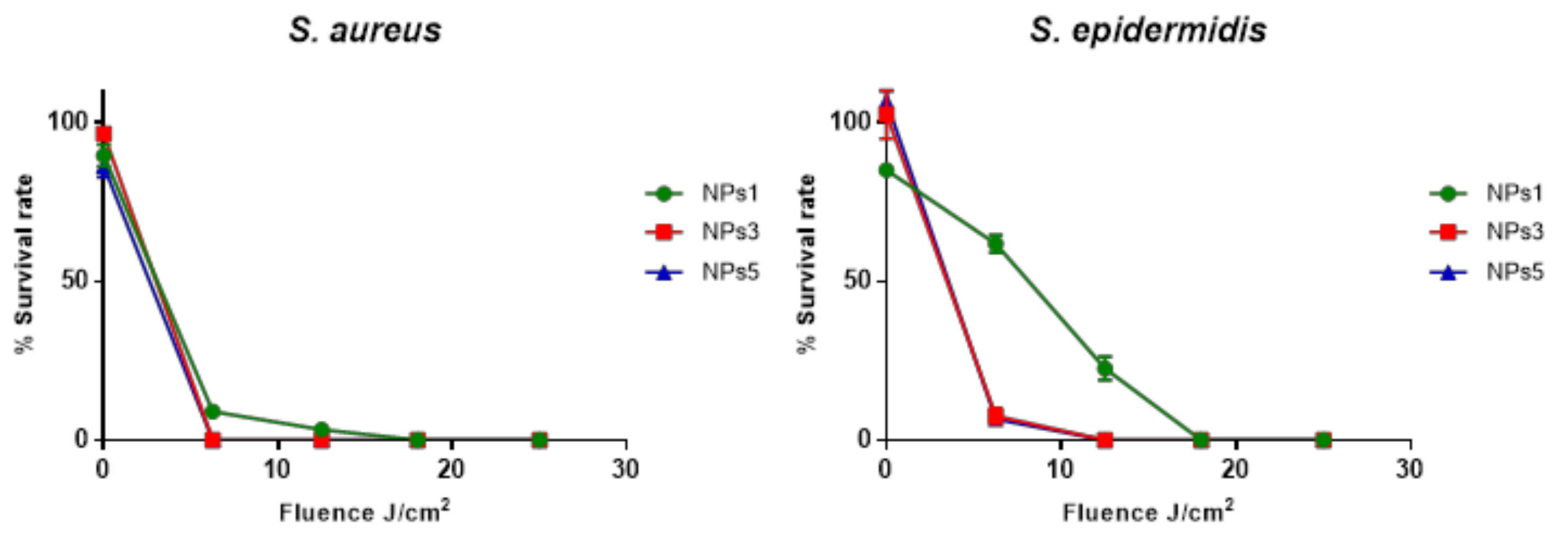
Photodynamic inactivation of *S. aureus* and *S. epidermidis* depending on the light dose.

The use of high fluence rates of the exciting light can cause oxygen depletion and PS photobleaching. Therefore, low fluence rate is more clinically relevant. To observe the influence of light dose on the survival rate of bacteria, *S. aureus* and *S. epidermidis* were directly put in contact with the most active NPs (**NPs 1**, **3** and **5**) in PBS (Figure 1). After an incubation period of 4 h in the dark in the different solutions, the washed bacteria were irradiated by the same device used previously. Bacterial concentration and survival rate were monitored following the increasing light dose. For each strain, three independent experiments were performed.

**NPs3** and **5** showed the best activity against these two bacterial strains since a low light dose (6.25 J/cm^2^) is sufficient to induce a reduction of up to three log of the survival rate. For **NPs 1**, a light dose of 18.75 J/cm^2^ is necessary to induce a complete eradication of bacterial strains.

**Figure 2:**
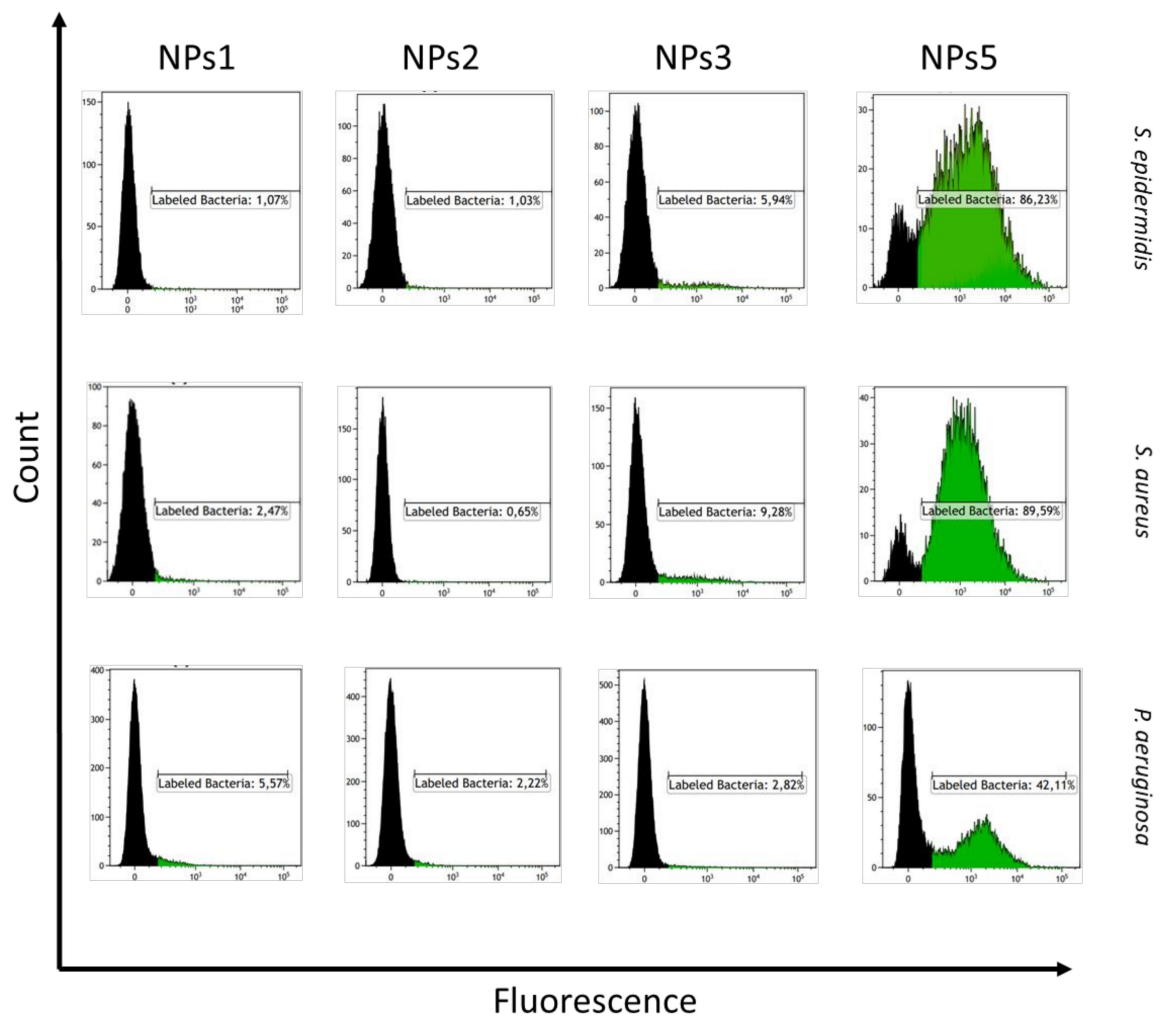
Flow cytometry analyses. Cytofluorometric profiles representing the distribution of bacterial cells labeled with NPs.

To understand more the results observed from the bacteria viability assays, flow cytometry experiments were performed to compare the interaction of NPs with bacteria (Figure 2). The two Gram-positive strains and one Gram-negative strain (*P. aeruginosa*) were incubated with the solutions of NPs (**NPs1**, **2**, **3** and **5**) at 10 µM. After 4 h of incubation in the dark at 37 °C, the bacteria were washed with PBS by centrifugation and the washed bacteria were resuspended with 500 µL of PBS. Fluorescence emissions were analyzed with a BD FACS Aria III cell sorter. Histograms were used to determine the percentage of labeled bacteria with NPs. These percentages were compared with the percentage obtained with the untreated bacteria used as a reference. **NPs1** and **NPs2** showed weak to no interaction with bacteria. These results are consistent with the low or lack of photobactericidal activity of **NPs1** and **NPs2** observed above. Although **NPs3** showed a photobactericidal activity, a weak interaction is observed with these NPs. Concerning **NPs5**, a remarkable fluorescence is obtained after incubation of these NPs with bacteria. This high fluorescence indicates a strong interaction of NPs with bacteria. We can observe a difference between the two types of bacteria. A lower interaction is obtained with the Gram-negative strain. However, it must be pointed out that flow cytometry only gives an idea of possible interaction between NPs and bacteria but does not give information about the uptake. This difference of interaction was highlighted in numerous studies that discuss the fundamental difference in susceptibility to aPDT between Gram-positive and Gram-negative due to the difference of the structure of the bacterial cell walls [40,41,42]. Gram-positive bacteria possess a thick cell wall (20–80 nm) of peptidoglycan as outer shell of the cell. In contrast, Gram-negative bacteria have a relatively thin (<10 nm) layer of cell wall composed of peptidoglycan but harbor an additional outer membrane made of layer of lipopolysaccharide [43]. These differences in the cell envelope confer different properties to the cell. One would have expected **NPs1** and **NPs2** to have the best interaction with bacteria, and hence photobacterial activity, because of their positive zeta potentials, especially **NPs1** which, in addition, is more hydrophobic. Indeed, the presence of a positive zeta potential should favor the electrostatic interaction between the particles and Gram-negative and Gram-positive bacteria whose membranes are negatively charged. However, the microstructure of **RuPLA** nanoconjugates seems to play an important role in their interaction with bacteria, and hence in their photobactericidal activity. More work needs to be done to have a complete understanding of the role of polymer microstructure on the interaction with bacteria.

## 4. Conclusions

In this study, we were able to obtain a modest photobactericidal activity on Gram-positive bacterial strains from a ruthenium(II) polypyridyl complex **RuOH** that could not, in its initial form, inactivate bacterial cells in the dark and under light irradiation. This has been made possible by preparing **RuPLA** nanoconjugates where **RuOH** is conjugated to the chain-end of PLA, an FDA approved biodegradable and biocompatible hydrophobic polymer. Depending on the microstructure and molecular weights of **RuPLA** nanoconjugates, differences in the photobacterial activity and interaction with bacteria have been evidenced with the best results obtained with the stereocomplex nanoparticles. Even though, modest photobactericidal activity has been observed, these results emphasize the potential of Ru(II) polypyridyl complexes if coupled with appropriate nanomaterials for aPDT. We are currently investigating the encapsulation of other Ru(II) complexes that absorb at higher wavelengths. These results will be published in due course.

## Figures and Tables

**Table 1:** Macromolecular and colloidal characterizations of **Ru-PLA** nanoconjugates.

Entry	Tacticity	*M*_n,NMR_ [a] (g/mol)	%RuOH [b] wt%	NPs	*D*_z_ ± SD [c] (nm)	PdI ± SD [c]	ζ (mV) [c]
**P1**	Atactic	7000	15	**NPs1**	149.7 ± 0.451	0.117 ± 0.005	33.4 ± 2.14
**P2**	Atactic	4000	25	**NPs2**	128.1 ± 0.737	0.171 ± 0.011	31.2 ± 2.22
**P3**	Isotactic	4000	25	**NPs3**	116.2 ± 0.551	0.167 ± 0.004	4.26 ± 0.401
**P4**	Isotactic	4000	25				
**P5**	Atactic	2000	53	**NPs4**	286.2 ± 3.10	0.173 ± 0.011	N.D.
**P6**	Isotactic	7000	15	**NPs5**	177.3 ± 1.55	0.183 ± 0.011	10.9 ± 0.231
**P7**	Isotactic	7000	15				

[a] *M*_n,NMR_ were calculated by ^1^H NMR spectroscopy in CD_3_CN. [b] Calculated according to (M(**RuOH**)/*M*_n,NMR_) × 100 with M(**RuOH**) = 1015.7 g/mol. [c] Determined by DLS as an average of three measurements, values given with standard deviation (SD).

**Table 2:** Minimum inhibitory concentration (MIC) and minimum bactericidal concentration (MBC) (μM) for **RuOH** and NPs against *S. aureus* and *S. epidermidis*.

NPs	MIC (µM)				MBC (µM)			
	*S. aureus*		*S. epidermidis*		*S. aureus*		*S. epidermidis*	
	Light	Dark	Light	Dark	Light	Dark	Light	Dark
**RuOH**	>50	>50	>50	>50	>50	>50	>50	>50
**NPs1**	25	>50	25	>50	>50	>50	25	>50
**NPs2**	50	>50	50	>50	>50	>50	50	>50
**NPs3**	25	>50	12.5	>50	50	>50	12.5	>50
**NPs4**	>50	>50	N.D.	N.D.	>50	>50	N.D.	N.D.
**NPs5**	12.5	>50	12.5	>50	25	>50	12.5	>50

MIC and MBC values have resulted from 3 independent experiments.

## Supplementary Materials

The following are available online at https://www.mdpi.com/1999-4923/12/10/961/s1, Figure S1: Reproducibility of the size distribution of **NPs1** by preparation of three independent batches, each measured three times, Figure S2: Reproducibility of the size distribution of **NPs2** by preparation of three independent batches, each measured three times, Figure S3: Reproducibility of the size distribution of **NPs3** by preparation of three independent batches, each measured three times, Figure S4: Reproducibility of the size distribution of **NPs5** by preparation of three independent batches, each measured three times.
