# Supplementary material for: Encapsulation of a Ru(II) Polypyridyl Complex into Polylactide Nanoparticles for Antimicrobial Photodynamic Therapy
Source: Pharmaceutics. 2020 Oct 13;12(10):961. doi: 10.3390/pharmaceutics12100961 (PMC7602071; doi:10.3390/pharmaceutics12100961)
Supplement: Supplementary file 1 [file pharmaceutics-12-00961-s001.pdf]

Supplementary Materials: Encapsulation of a Ru(II) Polypyridyl Complex into Polylactide Nanoparticles for Antimicrobial Photodynamic Therapy

Nancy Soliman, Vincent Sol, Tan-Sothea Ouk, Christophe M. Thomas and Gilles Gasser

			Size (d.nm):	% Intensity:	St Dev (d.nm):
Z-Average (d.nm):	155,0	Peak 1:	178,5	100,0	67,10
PdI:	0,125	Peak 2:	0,000	0,0	0,000
Intercept:	0,920	Peak 3:	0,000	0,0	0,000
Result quality :	Good				

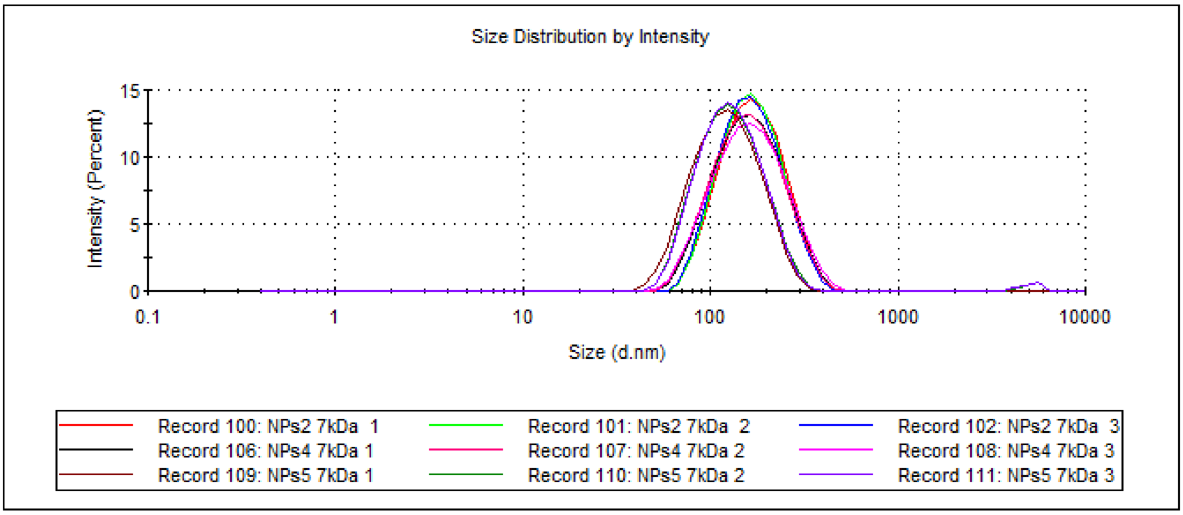

Figure S1. Reproducibility of the size distribution of **NPs1** by preparation of three independent batches, each measured three times.

			Size (d.nm):	% Intensity:	St Dev (d.nm):
Z-Average (d.nm):	118,8	Peak 1:	144,7	100,0	64,36
PdI:	0,177	Peak 2:	0,000	0,0	0,000
Intercept:	0,942	Peak 3:	0,000	0,0	0,000
Result quality :	Good				

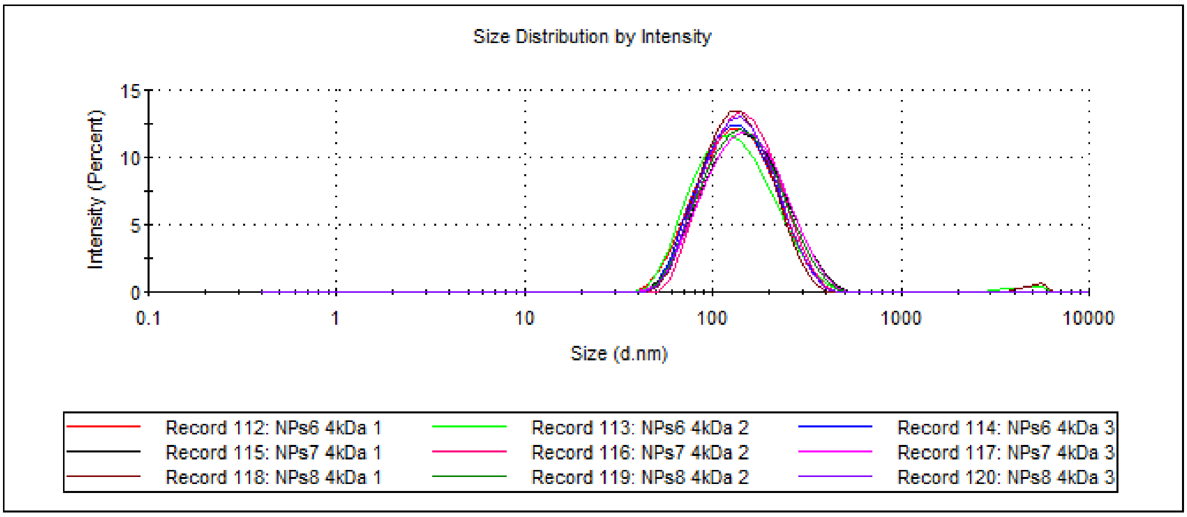

Figure S2. Reproducibility of the size distribution of **NPs2** by preparation of three independent batches, each measured three times.

			Size (d.nm):	% Intensity:	St Dev (d.nm):
Z-Average (d.nm):	156,0	Peak 1:	194,6	100,0	92,67
PdI:	0,200	Peak 2:	0,000	0,0	0,000
Intercept:	0,937	Peak 3:	0,000	0,0	0,000
Result quality :	Good				

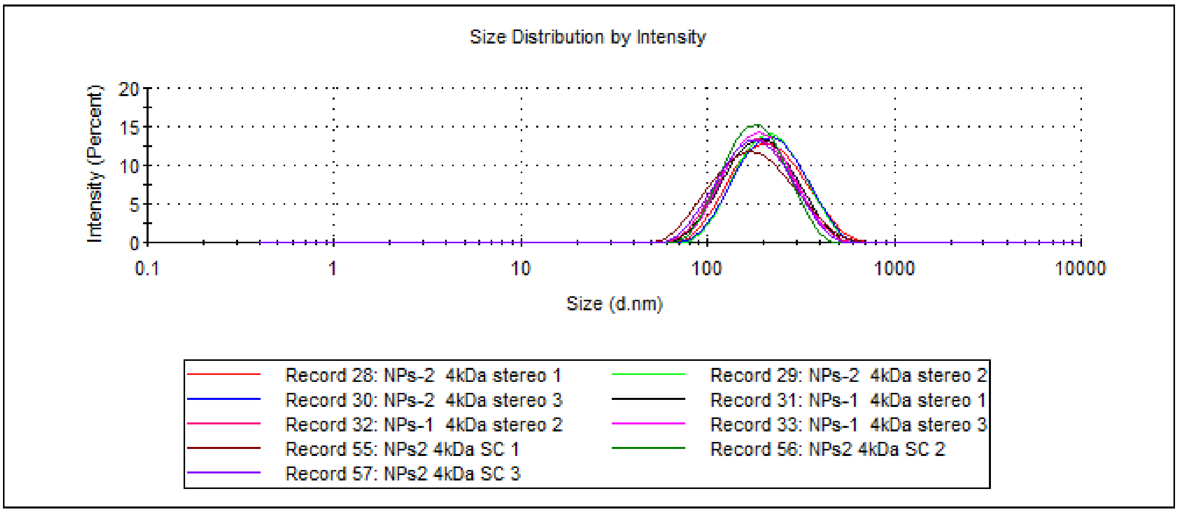

Figure S3. Reproducibility of the size distribution of **NPs3** by preparation of three independent batches, each measured three times.

			Size (d.nm):	% Intensity:	St Dev (d.nm):
Z-Average (d.nm):	178,8	Peak 1:	201,1	98,8	75,57
PdI:	0,190	Peak 2:	5120	1,2	530,2
Intercept:	0,934	Peak 3:	0,000	0,0	0,000
Result quality :	Good				

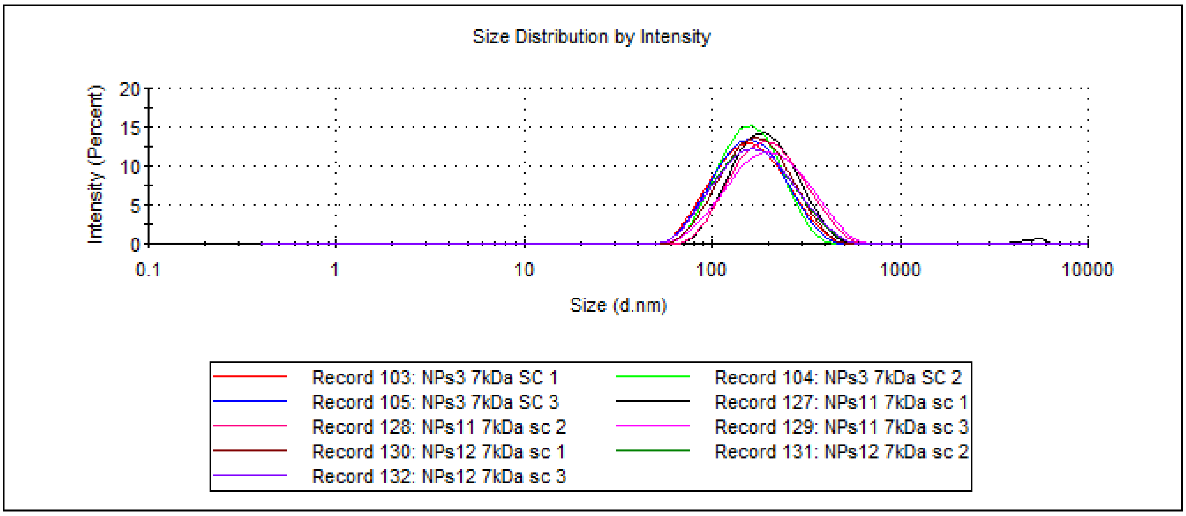

Figure S4. Reproducibility of the size distribution of **NPs5** by preparation of three independent batches, each measured three times.
